# A Novel nor@DHB Matrix for Direct Microbial Analysis in Lung Cancer Tissues

**DOI:** 10.1002/advs.202504038

**Published:** 2025-06-25

**Authors:** Liang Shan, Xin Xu, Lin Huang, Dan Li, Yiran Deng, Xiangfei Xue, Susu Guo, Yiman Huang, Xiao Zhang, Yongchun Yu, Lifang Ma, Kun Qian, Jiayi Wang

**Affiliations:** ^1^ Department of Clinical Laboratory Shanghai Chest Hospital Shanghai Jiao Tong University School of Medicine No. 241, West Huaihai Road Shanghai 200030 P. R. China; ^2^ Shanghai Institute of Thoracic oncology Shanghai Chest Hospital Shanghai Jiao Tong University School of Medicine No. 241, West Huaihai Road Shanghai 200030 P. R. China; ^3^ State Key Laboratory for Oncogenes and Related Genes School of Biomedical Engineering Institute of Medical Robotics and Med‐X Research Institute Shanghai Jiao Tong University No. 1954, Huashan Road Shanghai 200030 P. R. China

**Keywords:** diagnostic model, intratumoral microbiome, ionic liquid, lipid A, lung cancer, MALDI MS

## Abstract

Dynamic changes occurring in the lung microbiota can impact the initiation, progression, and prognosis of lung cancer (LC). Consequently, the development of suitable intratumoral microbiota analysis methods is crucial. Although matrix‐assisted laser desorption/ionization mass spectrometry (MALDI MS) involves straightforward operations and provides precise results, the “direct smear method” limits the identification of bacterial subspecies. Furthermore, the issue of inadequate quantification with MALDI MS renders it unsuitable for direct analysis of intratumoral bacteria. To address these challenges, a novel ionic liquid in this study is employed, called norharmane conjugated to 2,5‐dihydroxybenzoic acid (Nor@DHB) for the direct detection of intratumoral bacteria using MALDI MS. Because gram‐negative bacteria are dominant within cancer cells, lipid A is selected as the chemical fingerprint for bacterial identification. The results demonstrated that using Nor@DHB can enhance the lipid A signal by an order of magnitude and achieved a good linear relationship within a concentration range of 0.01–80 ng mL^−1^. Here, this method is successfully applied to the direct analysis of lipid A in actual clinical samples. Subsequent machine learning and nomogram models further confirmed the correlation between characteristic lipid A ions and LC patient clinicopathological features, which are further validated through both in vitro and in vivo experiments.

## Graphical abstract

Integrated matrix‐assisted laser desorption/ionization mass spectrometry workflow quantifies intratumoral microbes in lung cancer through lipopolysaccharide/lipid A extraction from biopsies. The novel ionic matrix called norharmane conjugated to 2,5‐dihydroxybenzoic acid enhances lipid A ionization, enabling direct detection of bacterial genera‐specific signatures. Synthetic lipid A calibration and clinical correlation in 50 patients validate this culture‐free method linking microbial load to cancer pathophysiology.

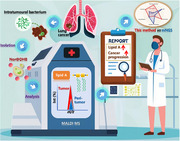

## 1 Introduction

As a mucosal organ directly connected to the outside environment, the lungs are exposed to a rich microbial flora. The dynamic changes of these microorganisms in both healthy and diseased states may have profound implications for the development of lung diseases, including lung cancer (LC).^[^ ^1^ ^]^ Disturbances in the microbiome, such as those caused by cigarette smoke, epithelial damage, or gene mutations, can allow pathogenic species to dominate the community or increase the virulence of other normally commensal microbes.^[^ ^1^ ^]^ Evidence of this has been demonstrated in patients with chronic lung disease who have more virulent forms of Mycobacterium tuberculosis, Pseudomonas aeruginosa, and Haemophilus influenzae.^[^ ^2^, ^3^, ^4^ ^]^ Compared with nontumor adjacent or tumor tissues, normal lung tissues display a lower alpha diversity of microbes. The alpha diversity of the microbiota within LC may also decrease with the enrichment of certain bacteria. Several bacteria are associated with chronic inflammation and the subsequent increased risk of LC. For example, Bacteroides fragilis can secrete endotoxins that cause DNA damage leading to mutations and cancer development.^[^ ^5^ ^]^ The activation of mitogen‐activated protein kinase (MAPK), mammalian target of rapamycin, tumor necrosis factor (TNF), and other pathways commonly observed in LC patients is associated with certain microbiota, such as *Veillonella* and *Streptococcus*.^[^ ^6^ ^]^ Furthermore, correlation have been found between the composition of the lung microbiota composition in LC and the pathological features of the disease. Thermus is increased in patients with advanced tumors, while Legionella is increased in patients with metastatic tumors. Additionally, Acidovorax exhibit higher abundance among the subset of squamous cell carcinoma cases with TP53 mutations.^[^ ^7^ ^]^ Surgical intervention is often required when treating LC. However, it is associated with a high incidence of postoperative pneumonia, which is primarily caused by gram‐negative bacteria.^[^ ^8^ ^]^

The intratumoral microbiome is closely associated with the initiation and progression of tumors, as well as with patient treatment outcomes, emphasizing the critical need for the development of suitable intratumoral microbiota analysis methods crucial. A robust microbial flora analysis within tumors currently hinges on deep sequencing of universal marker gene amplicons or 16S rRNA gene sequencing.^[^ ^9^ ^]^ However, these methods entail the use of costly equipment and reagents, driving up detection expenses. Furthermore, the raw data produced using high‐throughput sequencing necessitates intricate bioinformatics processing to yield meaningful insights, thereby augmenting the complexity and time required for result interpretating the results.^[^ ^10^ ^]^ Compared with sequencing technology, microbial identification using matrix‐assisted laser desorption/ionization mass spectrometry (MALDI MS) boasts simple operational procedures and accurate, reliable results, representing the most mature application of MALDI MS currently. Currently, MALDI MS combined with machine learning has emerged as a cutting‐edge technology in the field of bacterial analysis, demonstrating immense potential in enhancing identification speed and accuracy. For instance, machine learning algorithms, such as support vector machine, Random Forest, Logistic Regression, and CatBoost can automatically identify critical peaks in MALDI MS spectra, eliminate noise interference, and improve the discrimination capacity for closely related species.^[^ ^11^ ^]^ This combination is particularly valuable for classifying clinically significant and drug‐resistant bacteria, including *Staphylococcus aureus*, *Escherichia coli*, and *Klebsiella pneumoniae*.^[^ ^12^, ^13^ ^]^ Furthermore, machine learning compensates for the limitations of traditional methods through pattern recognition, enabling precise differentiation of strains that are easily confused by conventional biochemical identification, such as *Enterococcus faecium* and *Enterococcus lactis*.^[^ ^14^, ^15^ ^]^ By continuously learning and optimizing models, the capability of MALDI‐MS to identify rare strains and novel pathogens can be further enhanced.^[^ ^16^ ^]^ The clinically adopted “direct smear method” requires bacterial isolation and cultivation prior to MALDI MS analysis, which significantly restricts the identification of bacterial subspecies.^[^ ^17^ ^]^ Many of these microorganisms remain unidentifiable, often because of their rarity or difficulty in cultivation, coupled with unique cellular structures differing from conventional microorganisms, necessitating specialized preprocessing. For example, pathogenic mycobacteria possess a prolonged growth cycle ranging from 6 weeks to 5 months. The accuracy of MALDI MS in identifying these mycobacteria is impacted by various specimen preprocessing factors, such as the growth conditions, cell viability, and protein extraction techniques, ultimately increasing the costs and workload for diagnostic laboratories.^[^ ^18^ ^]^ For these reason, we chose to explore another alternative method that allows for the direct detection of intratumoral bacteria without the need for any isolation or purification steps.

Gram‐negative bacteria are the dominant bacterial species within cancer cells, whereas gram‐positive bacteria are rarely detected in either cancer cells or immune cells.^[^ ^19^ ^]^ Lipopolysaccharide (LPS or endotoxin), composed of the O‐antigen, the oligosaccharide core and the lipid A, is the major surface glycolipid located in the outer membrane of gram‐negative bacteria. Lipid A is the membrane‐forming moiety anchoring LPS within the membrane. As the most structurally conserved region of LPS. Lipid A is the Toll‐like receptor 4 (TLR4)‐activating component that triggers the early innate immune response to defend against gram‐negative bacterial infection.^[^ ^20^ ^]^ Previously, the use of lipid A was proposed for phenotyping bacteria, as they exhibited immense diversity in the arrangement of fatty acyl side chains and sugar‐associated functional groups. Extracts of *Klebsiella pneumoniae*, *Acinetobacter baumannii*, *Pseudomonas aeruginosa*, and *Enterobacter spp* were analyzed by MALDI MS to create a library of lipid A mass spectra, enabling direct identification of pathogens without culture.^[^ ^21^ ^]^ Consequently, it was hypothesized that lipid A represented a novel chemical fingerprint, which could potentially be used to identify intratumoral bacteria via MALDI MS in a manner analogous to that of bacterial proteins.

**Figure 1:**
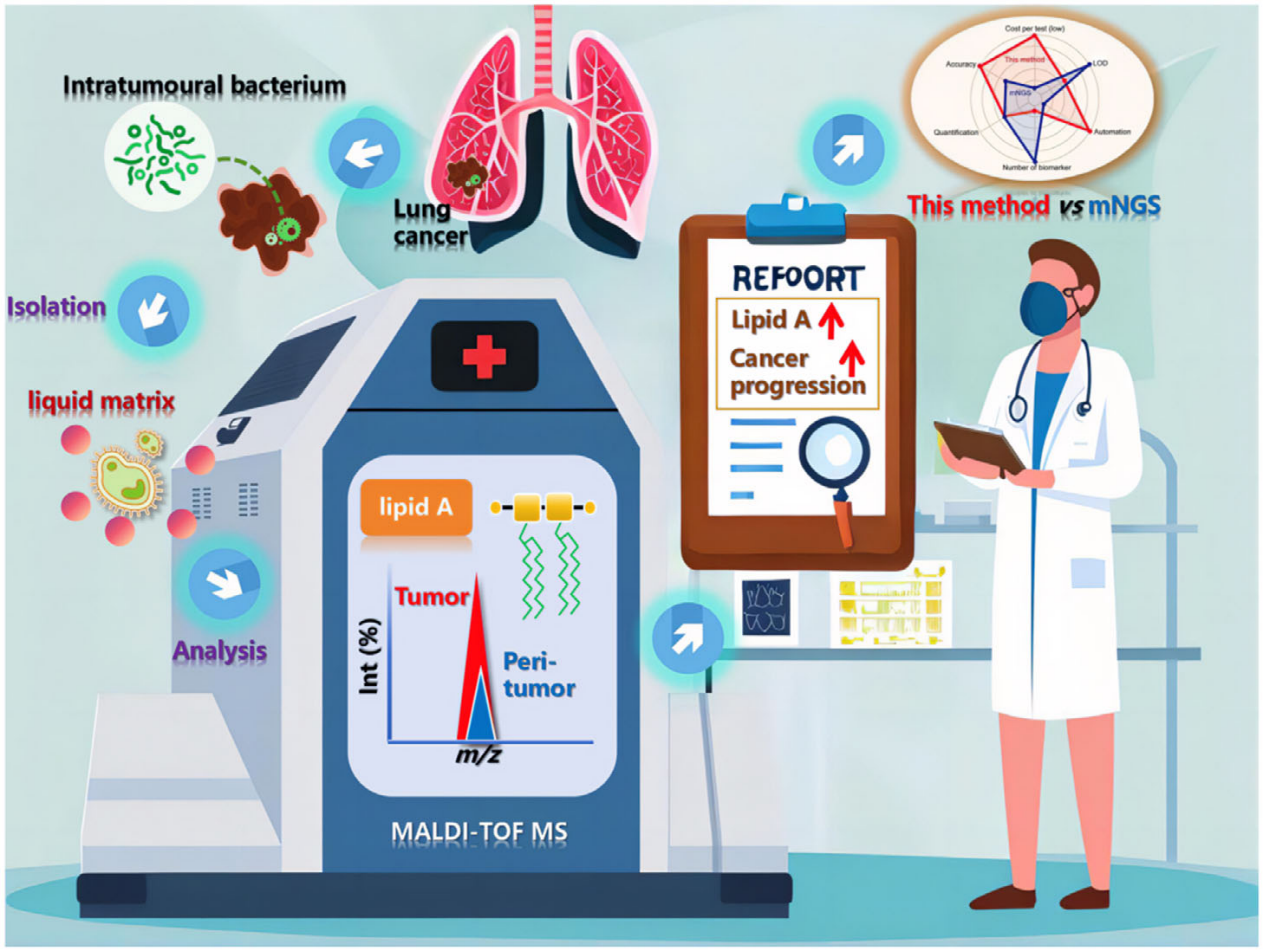
Research approach for the establishment of a direct, rapid, and highly sensitive method to quantify intratumoral microorganisms. This figure illustrates the integrated workflow developed to characterize and quantify intratumoral microbial communities in lung cancer using MALDI MS. The approach begins with the extraction of lipopolysaccharide from frozen lung tumor biopsies. A novel ionic liquid matrix, Nor@DHB—comprising Norharmane (Nor) conjugated to 2,5‐dihydroxybenzoic acid (DHB)—is then applied to enhance ionization efficiency of lipid A, a conserved bacterial membrane component. Direct MALDI MS analysis of crude extracts enables rapid profiling of lipid A species, with peaks annotated to specific bacterial genera. Quantitative validation is achieved through calibration curves of synthetic lipid A standards and correlation of the microbial load with clinical parameter in a cohort of 50 patients. This approach enables rapid, culture‐independent microbial quantification with direct relevance to lung cancer pathophysiology.

In MALDI experiments, 2,5‐Dihydroxybenzoic acid (DHB) is commonly used as a matrix for lipid analysis in positive‐ion mode, whereas 9‐Aminoacridine (9‐AA) is employed in negative‐ion mode. Hence, neither can function as a universal matrix for bacterial lipid analysis.^[^ ^22^ ^]^ Furthermore, tumor tissues typically harbor several thousand colony‐forming units (CFUs), yielding LPS quantities as minute as picograms, falling beneath the limit of detection (LOD) of both DHB and 9‐AA. Presently, the necessary amounts for lipid A are 1 ng and 100 pg, respectively.^[^ ^19^, ^22^ ^]^ Norharmane (Nor) is an indole alkaloid molecule, which serves as an exterior probe for photoinduced proton transfer as well as a biological photosensitizer. Nor was first reported as a matrix substance for MALDI in 1998.^[^ ^23^ ^]^ In MALDI experiments, the two functional centers of Nor, pyrrole, and pyridine nitrogen, can simultaneously enhance the detection of lipids and phospholipids simultaneously in both positive and negative ion modes. This characteristic may be related to how its pyrrole and pyridine nitrogen functional centers behave under different polarities. Nor has been used for better endotoxin detection in Francisella novicida‐infected tissues, and improving lipid A LOD to picogram levels.^[^ ^24^ ^]^ Recently, Nor has been successfully applied in MALDI MS imaging for lipid profiles in animal brains^[^ ^25^ ^]^ and tumors.^[^ ^26^ ^]^ Therefore, we speculate that Nor will be an ideal MALDI matrix for the direct analysis of lipid A derived from intratumoral microorganisms. To further evaluate the diagnostic value of bacterial lipid A in LC, a quantitative analysis of lipid A is needed. However, quantifying lipid A using MALDI MS remains challenging due to sample inhomogeneities and “sweet spot” effects.^[^ ^27^, ^28^ ^]^ To address the issues mentioned above, we planned a study that involved preparing a novel room‐temperature ionic liquid matrix (ILM), mixed with an equimolar volume of DHB and Nor (Nor@DHB). The ILM exhibits numerous qualities that are advantageous for use as a MALDI matrix. Specifically, its low volatility and ability to dissolve complex organic molecules have proven to be beneficial for enhancing quantitative analysis by improving shot reproducibility and providing more reliable average molecular mass measurements.^[^ ^29^ ^]^ Furthermore, an ILM containing Nor have already been successfully used for analyzing low molecular weight carbohydrates.^[^ ^30^ ^]^ To further mitigate the impact of complex matrix effects, we incorporated a straightforward LPS extraction step prior to MALDI MS analysis on the collected tissue samples. Following extraction, the samples were mixed with Nor and DHB, then subsequently subjected to mass spectrometry for direct analysis. This approach allows for the direct detection of lipid A in complex LC tissues using MALDI MS. Our goal is to establish a direct, rapid, and highly sensitive quantitative method for intratumoral microorganisms, thereby providing a foundation for the diagnosis and treatment of LC (**Figure** **1**).

## 2 Results and Discussion

### 2.1 Characteristic Microbial Types Exist Within LC Tissues

**Figure 2:**
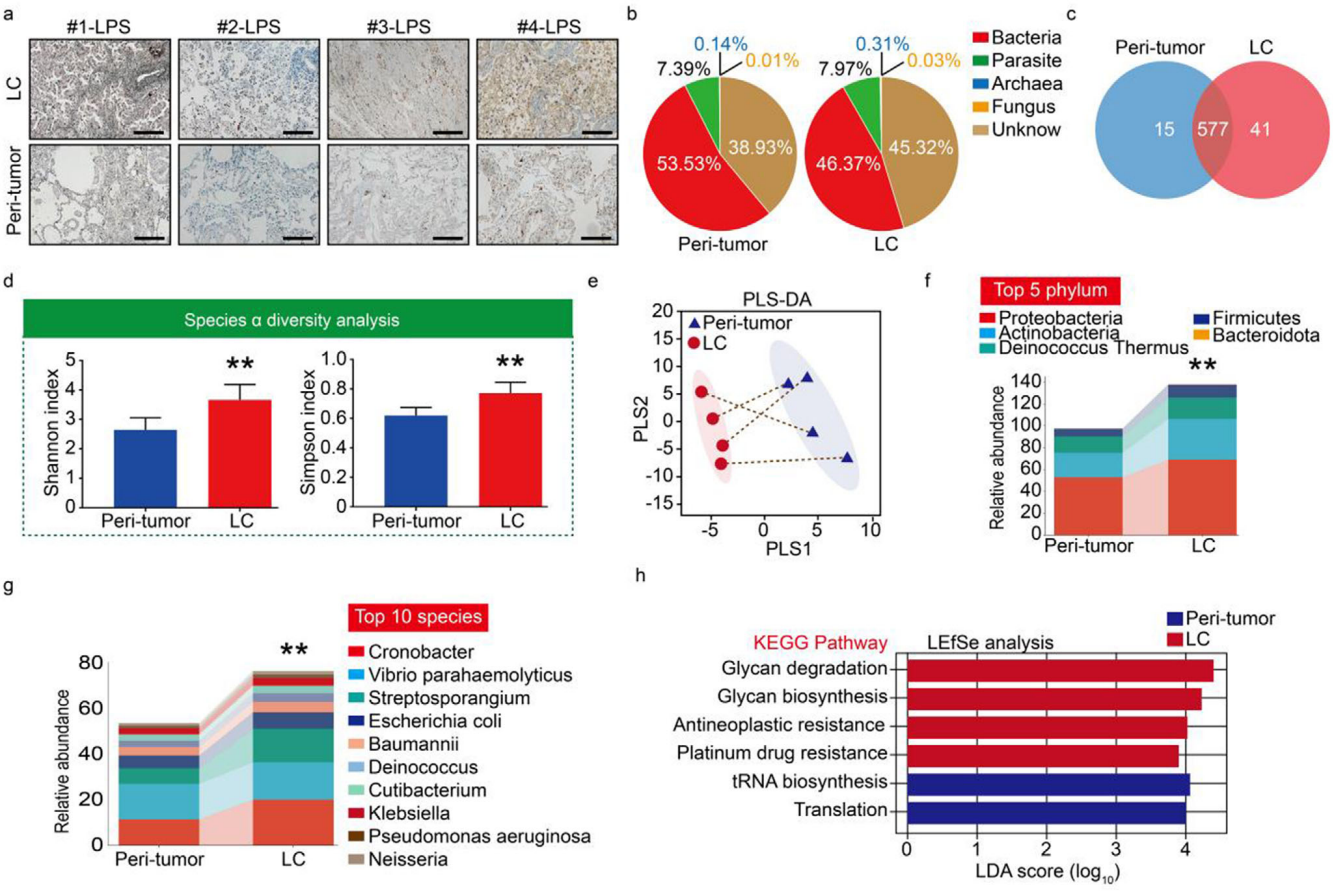
The presence of abundant microorganisms in LC tissues. a) LPS levels in LC and peri‐tumor tissues as determined by immunohistochemical analysis. b,c) Distribution of different types of microorganisms in LC and peri‐tumor tissues detected by mNGS (*n* = 4). d) Analysis of alpha diversity of microorganisms in LC and peri‐tumor tissues, **P < 0.01. e) Analysis of beta diversity of microorganisms in LC and peri‐tumor tissues. f) Differences in microbial abundance at the phylum level in the LC and peri‐tumor tissues, *P < 0.05. g) Differences in microbial abundance at the species level in the LC and peri‐tumor tissues, **P < 0.01. h) Histograms showing KEGG enrichment of differential microorganisms.

To investigate the presence of bacteria within LC tissues, we performed histological staining and metagenomic next‐generation sequencing (mNGS) to analyze four pairs of LC tissues and their peri‐tumor tissues. First, LPS could be detected in both the LC and adjacent noncancerous tissues, with the LPS abundance found to be significantly higher in LC than in the peri‐tumor tissues (**Figure** **2**a; and Figure S1, Supporting Information). Because this study focused on the direct analysis of gram‐negative bacterial lipid A within LC tissues, lipoteichoic acid (LTA) staining was not performed. According to a report published in *Science* in 2020, LTA was primarily detected in melanomas and largely absent in other tumor types, including LC.^[^ ^19^ ^]^ Next, mNGS results revealed that a diverse range of microorganisms was present in the LC tissues, with bacteria dominating (≈50%), followed by fungi (7%), while unknown species accounted for about 40% (Figure 2b). Among all identified microorganisms, 577 species were present in both the LC and adjacent tissues, while 41 species were unique to the LC tissues and 15 were unique to the adjacent tissues. These data suggest that not only that abundant microbial types existed within LC, but also that there are potential differences in microbial characteristics between LC and adjacent tissues (Figure 2c). Considering the significant risk of contamination in samples with low biomass, environmental samples were used as a false‐positive control to exclude potential contamination in the mNGS experiment. Additionally, the LC and adjacent tissues were stored and processed under the same conditions, implying that the mNGS analysis results were not caused by exogenous contamination.

To identify microbial changes associated with LC, we first examined the ecological diversity within samples (alpha diversity) of nontumor adjacent and LC tissues. Specifically, using the Shannon index and Simpson index, which measures both the number (richness) and abundance (evenness) of species, a significantly increased alpha diversity was observed in the tumor tissues compared to the adjacent tissues (Figure 2d). We also investigated whether there were differences between samples using beta diversity. The results showed that microbial data obtained using mNGS could effectively differentiate between LC and adjacent tissues, demonstrating heterogeneity between the two groups (Figure 2e). Collectively, these data illustrate a trend of increasing diversity and richness associated with LC. The top five most abundant bacterial groups in the LC samples were Proteobacteria, Deinococcus‐Thermus, Actinobacteria, Firmicutes, and Bacteroidota (Figure 2f). These findings are consistent with those of Greathouse et al.^[^ ^7^ ^]^ At the genus and species levels, the bacterial flora within the LC tissues are predominantly composed of gram‐negative bacteria, such as *Vibrio*, *Cronobacter*, and *Klebsiella*, with an abundance of these bacteria is greater than that in the adjacent noncancerous tissues (Figure 2g; and Figure S2, Supporting Information). These gram‐negative bacteria colonizing within LC tissues are the main culprits of severe postoperative infections and may interact with CTC (circulating tumor cells) to contribute to the recurrence and metastasis of LC.^[^ ^8^ ^]^ Additionally, KEGG enrichment analysis revealed that these differentially abundant bacteria were significantly enriched in glycan degradation, glycan biosynthesis, and drug resistance, which are closely related to LC progression (Figure 2h; and Figure S3, Supporting Information). This further emphasizes the practical significance of developing effective analytical methods for intratumoral bacteria.

### 2.2 Methodological Validation

**Figure 3:**
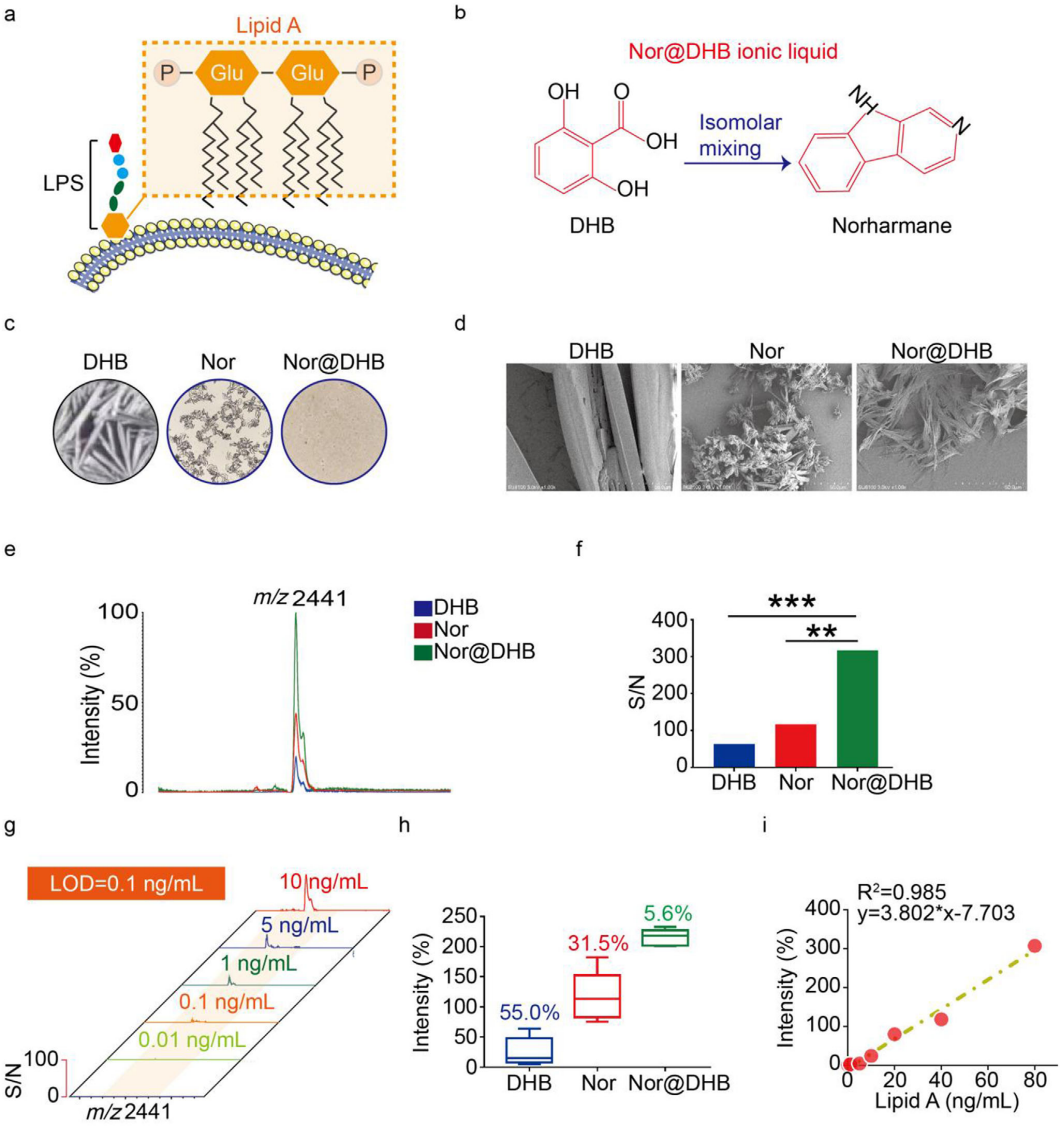
Methodological validation. a) Schematic diagram of the Lipid A structure on the surface of bacteria. b) Ionic liquid: Nor@DHB. c) Microscopic images of DHB, Nor and Nor@DHB. d) Scanning electron microscope images of DHB, Nor and Nor@DHB. Scale bar, 500 µm. e) Mass spectrometry of lipid A collected using DHB, Nor and Nor@DHB as the matrix. f) Statistical results of S/N collected using DHB, Nor and Nor@DHB as the matrix. g) MS of lipid A at 0.01, 0.1, 1, 5, and 10 ng mL^−1^ collected using Nor@DHB as the matrix. h) Repeatability analysis of lipid A ion signals collected using DHB, Nor or Nor@DHB as the matrix. i) Linear relationship between the signal intensity and the concentrations of lipid A collected using Nor@DHB as the matrix.

The lipid A structure is characterized by multiple fatty acyl residues linked to a D‐glucosamine disaccharide backbone, which is a relatively unique feature in nature. The hydroxyl groups of saturated 3‐hydroxy fatty acids hydroxyl groups of lipid A can be further acylated to form 3‐acylated fatty acids, a unique component of lipid A that aids in distinguishing different bacterial species. Additionally, the lipid A structure is stable and maintains good detectability. Direct detection of lipid A in complex LC samples using MALDI MS can yield reliable identification results even when the bacterial content is low^[^ ^31^ ^]^ (**Figure** **3**a). To enable the direct analysis of bacterial lipid A signals within LC tissues, a novel ILM called, Nor@DHB, was prepared by mixing DHB and Nor in an equimolar ratio (Figure 3b). Subsequently, lipid A standards derived from *Escherichia coli* were used as model analytes to investigate the analytical performance of Nor@DHB for lipid A. The Nor@DHB employed in this study efficiently dissolves lipid A, resulting in smooth and uniform spots on the MALDI plate. This contrasts the solid matrices DHB and Nor, which exhibit cracking, crystallization, and numerous inconsistencies (Figure 3c). At a 1000 × magnification, the crystal morphologies of the three matrix materials exhibit notable differences: DHB crystals predominantly feature coarse particles with uneven size distributions; Nor crystals form rough‐surfaced, dendritic clusters. In contrast, the Nor@DHB composite crystals display a unique filamentous structure with highly uniform diameters, smooth surfaces, and regular orientations (Figure 3d). Using Nor and Nor@DHB as matrix enhanced the signal‐to‐noise ratio (S/N) of the lipid A ion by approximately one order of magnitude compared to DHB, confirming the effectiveness of Nor for lipid analysis in negative ion mode. Furthermore, when Nor@DHB was used as the matrix, the signal response for lipid A was significantly increased compared with using the Nor (Figure 3e,f). The LOD for clearly distinguishing any peak as a signal from the noise was defined as an SNR greater than 3. The data shown in Figure 3g revealed that the S/N of the lipid A standard correlates with its concentration (ranging from 0.01 to 10 ng mL^−1^). The LOD was ≈0.1 ng mL^−1^ when Nor@DHB was used as the matrix, indicating exceptional sensitivity for lipid A analysis. During the MALDI process, the matrix must generate ions from the analyte of interest following laser desorption/ionization. Proton transfer reactions between the matrix and analyte can produce either positive or negative ions. In contrast to DHB, the base component of Nor@DHB plays a crucial role in facilitating proton transfer between the analyte and matrix. Upon laser irradiation, proton transfer occurs not from the acid moiety but from the corresponding salt, which significantly boosts sensitivity. Additionally, when DHB was used as a matrix, lipids detected in MALDI MS may form adduct ions with sodium or potassium, which can suppress the ion intensity and degrade the resolution. However, Nor@DHB eliminates the presence of sodium and potassium, thereby improving the ion intensity and resolution.^[^ ^32^ ^]^ These factors resulted in Nor@DHB having an increased sensitivity when used for lipid A analysis compared with using DHB or Nor individually.

Furthermore, to enable the quantitative analysis of bacterial lipid A in both LC and noncancerous tissues, excellent analytical sensitivity alone is insufficient. Previous studies have highlighted the potential benefits of using a liquid matrix in MALDI, including achieving a more homogeneous blend of analytes and matrix, enhancing reproducibility among spots, and facilitating more laser shots per spot from enhanced diffusion and sample flow. Compared with the control group, incorporating of Nor@DHB not only boosted the overall ion intensity but also improved the reproducibility in the MALDI MS analysis. Specifically, the coefficient of variation (CV) of the ion signals was reduced from 55.0% with DHB alone to 31.5% with Nor, which was further reduced to 5.6% with Nor@DHB (Figure 3h). Encouraged by these improvements in signal sensitivity and reproducibility, a strong linear relationship was observed within the concentration range of 0.1–80 ng mL^−1^ after adding Nor@DHB to the sample solution (*y* = 3.802*x−7.703, *R* ^2^ = 0.985). This suggested that Nor@DHB could be a valuable tool for lipid A quantitative analysis from LC‐associated bacteria using MALDI MS (Figure 3i).

### 2.3 The Bacterial Lipid A is Specifically High‐Expressed in LC Tissues

**Figure 4:**
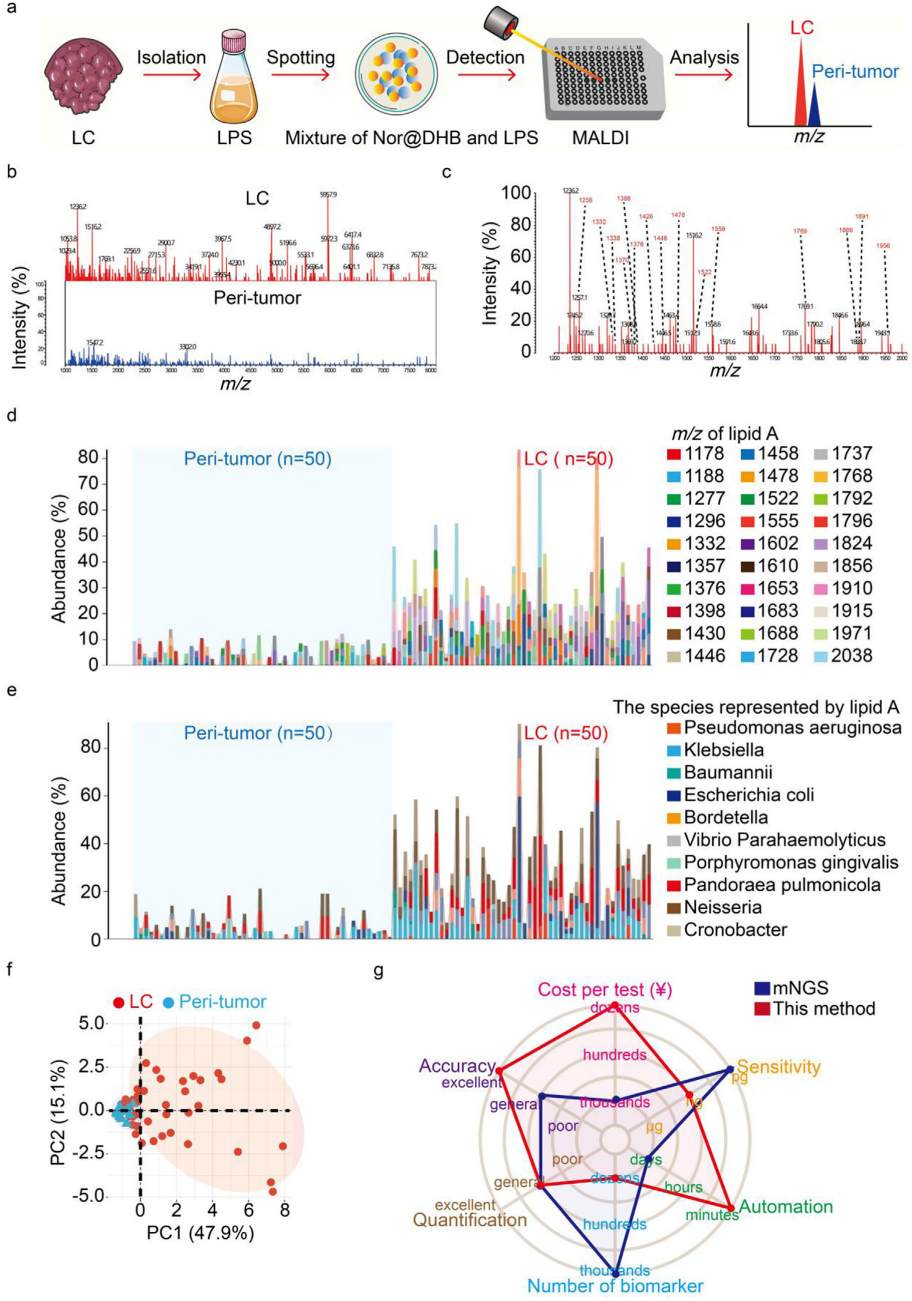
Bacterial lipid A levels are specifically elevated in LC tissues. a) Using Nor@DHB as the matrix can achieve simple and rapid MALDI MS analysis of intratumoral bacteria. b) Comparison of the spectra of LC and peri‐tumor tissues using Nor@DHB as the matrix. c) The lipid A ions detected in LC tissues. d,e) Differences in the abundance of lipid A ions and the bacteria they represents in the LC and peri‐tumor tissues. f) PCA clustering diagram of the 100 LC and their peri‐tumor tissue paris. g) The advantages and disadvantages of our new approach compared with mNGS.

During the MALDI MS analysis, highly abundance substances within the samples competitively attract charges, decreasing the ionization efficiency of less abundance analytes. Utilizing Nor@DHB as a matrix can notably boost the analytical performance for lipid A, but direct tissue homogenate analysis without extraction results in MALDI spectra dominated by high‐abundance tissue proteins (Figure S4, Supporting Information). Low‐input extraction methods for MALDI MS analysis of lipid A have been reported. The most widely employed method was developed by the Caroff's group, which uses a mixture of isobutyric acid and ammonium hydroxide to both solubilize and facilitate lipid A hydrolysis.^[^ ^33^ ^]^ Yang et al. also reported a method for on‐tissue derivatization of LPS for lipid A detection using MALDI MSI.^[^ ^34^ ^]^ Therefore, LPS extraction was performed on tumor tissues prior to analysis to enhance the response to bacterial lipid A signals. Then, the extract was blended with Nor@DHB, placed on the sample plate, and analyzed by MS. The entire process of this newly established method took ≈1 h to analyze dozens of tissue samples. However, the entire workflow of the MALDI MS analysis method using bacterial culture, from sample cultivation to obtaining the final identification results, takes roughly 20–30 h for common bacteria and yeast‐like fungi. For some microorganisms with slow growth rates, such as certain anaerobic bacteria, filamentous fungi, and slow‐growing mycobacteria, the entire process may require several days (**Figure** **4**a). To evaluate the clinical significance of lipid A in LC analysis, we further studied 50 pairs of LC and adjacent noncancerous tissues (Table S1, Supporting Information). Subsequently, tissue lysates were analyzed by MALDI MS within the low‐mass range. The results shown in Figure 4b,c revealed distinct differences in the lipid fingerprints between LC and peri‐tumor tissues. Because of individual variations, not all signal peaks could be identified, so 30 reliable peaks (belonging to 15 different strains) with ion intensities exceeding 10 were chosen for further analysis (Table S2 and Figure S5, Supporting Information). Like results obtained by mNGS, the MALDI MS data in LC revealed significantly increased in characteristic lipid A signals and the corresponding bacteria in LC, such as *Vibrio parahaemolyticus*, *Klebsiella pneumoniae*, *Acinetobacter baumannii*, *Neisseria species*, and *Cronobacter species*. These bacterial species were also determined by mNGS to be present at differential levels between LC and adjacent noncancerous tissues (Figure 4d,e). Moreover, high‐dimensional protein MS data were analyzed using principal component analysis (PCA), focusing on the first (47.9%) and second (15.1%) principal components for visualization. The PCA model could distinctly separate LC tissues from peri‐tumor tissues, highlighting the heterogeneity between the groups (Figure 4f). Finally, all matrices (including Nor and DHB used individually) exhibited relatively weak signals in the high molecular weight region, aligning with the inherent property of DHB or Nor being unsuitable for ionizing large molecules. The faintly discernible weak signals observed in this region may stem from trace contamination by high‐molecular‐weight proteins present in the tissue samples. This finding further reinforces the selective advantage of Nor@DHB within the target analytical range, where it minimizes matrix‐derived background interference while maintaining robust ionization efficiency for lipid A (Figure S6, Supporting Information).

Our rapid method can be used to analyze intratumoral bacteria directly from tissue samples without bacterial culture. However, because of MS limitations, only a limited number of abundant bacteria can be analyzed. Although mNGS boasts higher detection sensitivity and can theoretically identify all intratumoral bacteria with known genomes, it generates vast sequencing data that require complex bioinformatics methods and strong expertise to examine. Interpreting the results demands clinical experience and professional knowledge, potentially leading to varied conclusions and increased uncertainty. Our method offers more accurate bacterial characterization through analysis of the intratumoral bacterial lipid A *m/z* values. Furthermore, we found that Nor@DHB could enhance the MALDI matrix uniformity, enabling quantitative MALDI‐MS analysis, which aided the comparison of bacterial differences between the LC and peri‐tumoral tissues. Most importantly, compared with the high cost of mNGS technology (≈¥1200 per sample), our method would costs < ¥10 per sample, highlighting its noteworthy clinical applicability (Figure 4g).

### 2.4 Construction of a LC Diagnostic Model Using Lipid A

**Figure 5:**
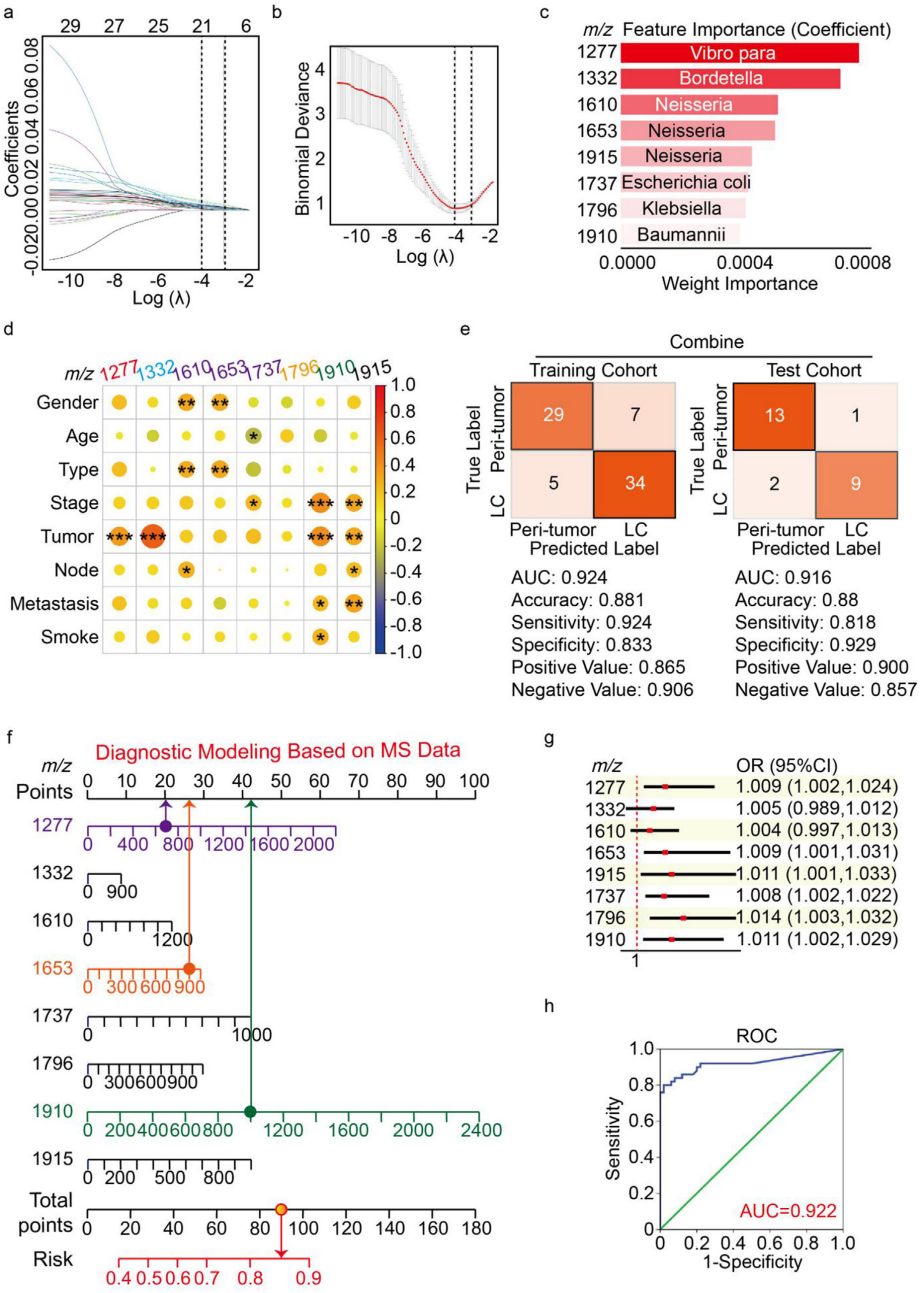
Construction of the diagnostic model. a,b) A coefficient profile plot was constructed against the log (lambda) sequence. c) Model influence factor importance analysis. d) Correlation analysis of intratumoral bacterial lipid A ions and LC pathology. e) The lipid A signals are used for LC and peri‐tumor classification through the machine learning confusion matrix. f) Nomogram predicting the diagnostic value of lipid A ions for LC. g,h) Forest plot and ROC used to assess Nomogram reliability.

Using the newly established intratumoral bacterial analysis method, the correlation between characteristic lipid A signals and LC pathology were further evaluated. The Lasso model, a regression method suitable for addressing multicollinearity issues, enables variable selection while estimating parameters. To further screen for suitable lipid A biomarkers, Lasso regression analysis was performed to select predictive variables from among 30 lipid A peaks. Ultimately, eight of the 30 variables were included in the predictive model, namely *m/z* 1277, 1332, 1610, 1653, 1915, 1737, 1796, and 1910 which had nonzero coefficients (**Figure** **5**a,b). The results in Figure 5c indicated the ranking of variable importance obtained from the analysis, with these eight variables will be used in subsequent modeling. The bacteria represented by the characteristic lipid A are also annotated in Figure 5c. Moreover, we found that the signal intensities of the eight lipid A peaks remained significantly associated with the stage and TNM classification of LC patients (Figure 5d). Using the independent variables selected with the Lasso model, a machine learning approach was used to identify characteristic bacteria that have a significant impact on LC progression. To optimize the parameters for the training model and assess its performance, 15.00% of the total samples were randomly selected as the test set, while the remaining samples were used as the training set for twofold cross‐validation. Although the area under curve (AUC) results for the individual variables in the model were not satisfactory, the AUC values for the combined diagnosis of the eight variables were 0.924 and 0.916 in the training and test sets, respectively. These data suggested that this combination of biomarkers has clinical significance for LC progression (Figure 5e; and Figure S7, Supporting Information). To evaluate the reliability of the machine learning strategy, the classification results using these lipid A biomarker combinations showed relatively high accuracy in distinguishing between LC and peri‐tumor tissues (Figures S8–S10, Supporting Information). Furthermore, a nomogram was generated to quantitatively predict the progression probability of LC based on the Lasso regression model (Figure 5f). For example, using the nomogram model, a LC with an *m/z* 1277 ion intensity of 620, an *m/z* 1653 ion intensity of 900, and an *m/z* 1910 ion intensity of 1000 had an estimated 85% risk of a higher degree of malignancy. These lipid A signals respectively indicate high abundances of *Vibro para*, *Neisseria*, and *Baumannii* colonization in the LC tissues (Figure 5f). Similarly, the validity evaluation of the nomogram model validity evaluation using binary logistic regression demonstrated that the model has excellent discriminatory ability between LC and adjacent noncancerous tissues, with an AUC value of 0.922 (Figure 5g,h, Supporting Information).

By amplifying lipid A signal, Nor@DHB can enable the detection of low‐abundance gram‐negative bacteria, which are often masked by host‐derived molecules in tumor tissues. The linear dynamic range allows for the absolute quantification of lipid A which is critical for correlating the bacterial load with clinical outcomes. This innovation overcomes the limitations of traditional bacterial culture methods, which are often time‐consuming, labor‐intensive, and cannot accurately represent the in situ microbial composition. Our method allows for the detailed characterization of the intratumoral microbiota, which can provide insights into the correlation between the microbiome and tumor progression. By identifying specific bacterial species or strains that are associated with different stages of lung cancer, we may be able to better understand the mechanisms underlying tumor development and progression. From a clinical perspective, LC surgery remains a necessary treatment approach. However, severe infection is a common complication following lobectomy and significantly reduces the 5‐year overall survival rate. Postoperative pneumonia is caused by gram‐negative bacteria, with an incidence rate as high as 25%.^[^ ^35^ ^]^ The information gained from our method could also inform the development of personalized treatment strategies for lung cancer patients. For example, if certain bacterial species are found to be associated with poor prognosis or resistance to specific therapies, targeting these bacteria or modulating the microbiota could potentially improve treatment outcomes.

### 2.5 Functional Validation

**Figure 6:**
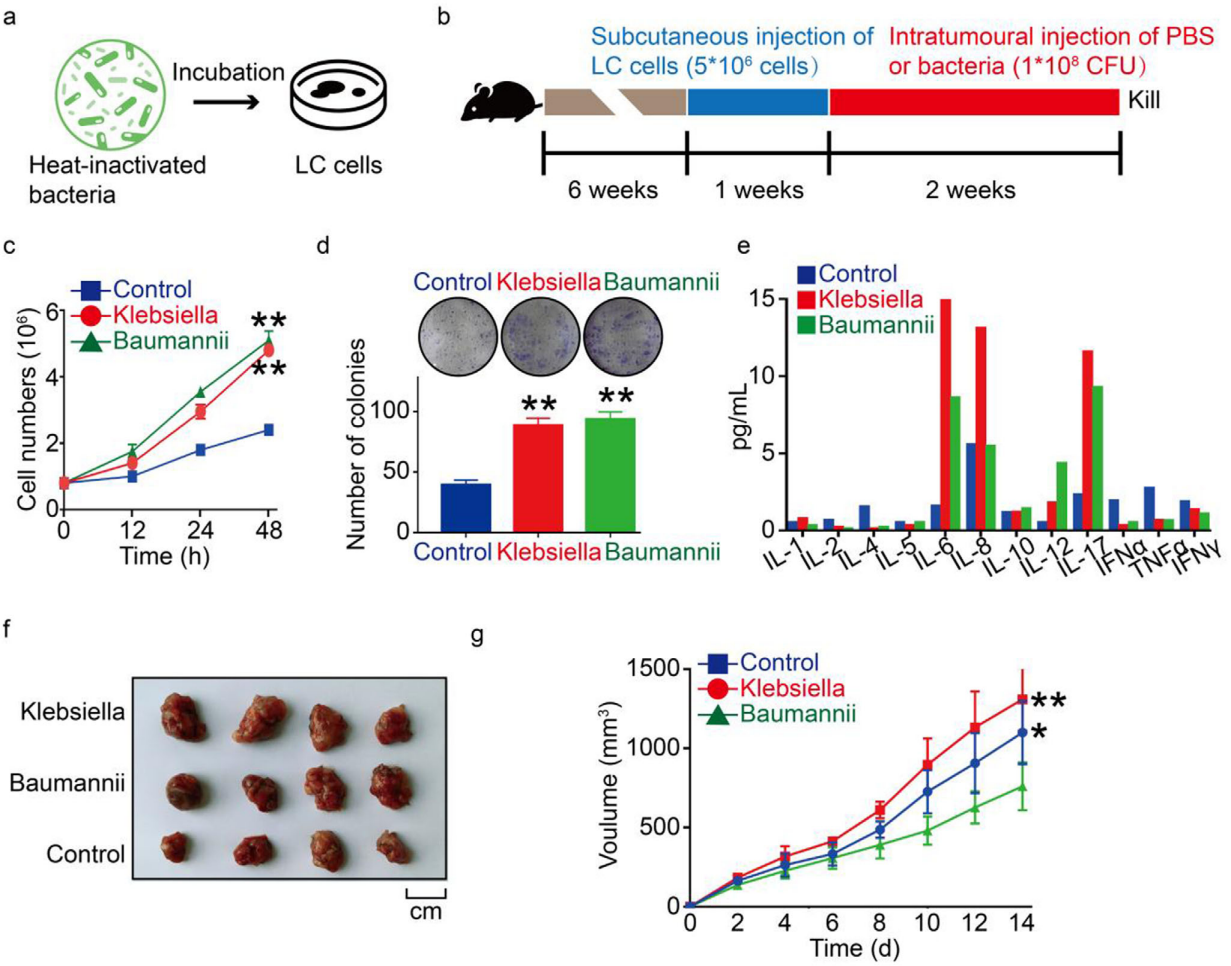
Functional validation. a,b) In vitro and in vivo experimental design. c) Cell viability of A549 cells treated with *Klebsiella* and *Baumannii*, **P < 0.01. d) Colony formation was performed to test the effects of *Klebsiella* and *Baumannii* on A549 cells, **P < 0.01. e) Cytokine levels in the A549 cells treated with *Klebsiella* and *Baumannii*. f,g) Representative images and volume of tumors treated with *Klebsiella* and *Baumannii*, *P < 0.05,**P < 0.01.

Lung epithelial cells form a mechanical barrier against microbial invasion, activating innate immune responses through the expression of TLRs. TLR4 is primarily activated by binding to LPS derived from the cell wall of gram‐negative bacteria, with lipid A serving as the toxic and biologically active center of LPS. Activated TLR4 further stimulates intracellular signaling pathways such as MAPK and nuclear factor kappa‐B (NF‐κB), leading to a series of pathological responses in host cells. Similar to lung epithelial cells, both LC cells and circulating tumor cells express functional TLRs, which can directly bind to bacteria, contributing to severe postoperative infections or tumor progression and metastasis. To further verify the reliability of the newly established method and the impact of Gram‐negative bacteria on tumor progression, heat‐inactivated *Klebsiella* and *Baumannii* species were selected for in vivo and in vitro functional validation, instead of purified bacterial antigens. Heat‐inactivated bacteria are increasingly utilized for the preliminary evaluation of bacterial‐host interactions, owing to their convenience, reproducibility, and diminished biosafety risks. These advantages make them especially well‐suited for experimental paradigms that involve long‐term feeding, such as tumor models, and they have emerged as a widely acknowledged research tool^[^ ^8^, ^36^ ^]^ (**Figure** **6**a,b). First, LC cells were treated with *Klebsiella* and *Baumannii*, and the results indicated that the proliferative effect of bacteria was time‐dependent (Figure 6c). Additionally, compared to the control group, *Klebsiella* and *Baumannii* effectively induced the formation of colonies derived from LC cells (Figure 6d). LPS can bind to TLR4 on the surface of cancer cells through its lipid A structure, and further activate downstream NF‐κB through MyD88‐dependent and ‐independent pathways. This stimulates the production of cytokines, such as TNF‐α, interleukin ‐ 1β (IL‐1β), IL‐6, and IL‐17, which in turn promote tumorigenesis through a chronic inflammation‐repair mechanism.^[^ ^37^, ^38^ ^]^ Our results also showed that LC cells cocultured with Klebsiella and Baumannii secreted significantly more IL‐6, IL‐8, and IL‐17 compared to the control group (Figure 6e). The various cytokines produced by LPS have different signaling functions. According to the classic “multiple hits” theory, the immune damage to cells mediated by the cytokine IL‐1β and the direct cytotoxic effect of TNF‐α are considered the first hits of innate immunity on cells.^[^ ^39^ ^]^ In addition to the aforementioned immune damage, IL‐6 can activate the downstream Janus kinase/signal transducer and activator of transcription, inhibiting Fas‐mediated apoptosis by inactivating Caspases and downregulating reactive oxygen species, thereby exerting a regenerative and repair effect on cells.^[^ ^40^ ^]^ The secretion of IL‐8 and IL‐17 can recruit tumor‐associated macrophages or promote the M2 polarization of macrophages, further altering the tumor microenvironment and accelerating the progression of lung cancer.^[^ ^41^ ^]^ Furthermore, in subcutaneous tumor‐bearing BALB/c nude mice, tumor tissue grew faster in both the *Klebsiella* and *Baumannii* treated groups compared to the control group. These results suggest that gram‐negative bacteria have the ability to promote the proliferation of LC cells (Figure 6f,g).

Although the Nor@DHB–MALDI MS method developed in this study has significantly enhanced the detection capability of intratumoral microorganisms, it still has the following key limitations: First, the method's core reliance on lipid A, a specific marker of Gram‐negative bacteria, restricts its ability to detect critical microbial components, such as Gram‐positive bacteria, fungi, and archaea. This may result in the omission of a complete profile of potentially carcinogenic microorganisms. Second, due to the technical targeting limitations, the current strategy struggles to elucidate the dynamic interactions within microbial communities (e.g., the synergistic regulation of the tumor microenvironment by bacterial‐fungal symbiotes), while these interactions may be important mechanisms driving the progression of LC. In addition, the cohort sample size is insufficient, and the population diversity is lacking. Moreover, the existing data lack longitudinal tracking to evaluate their predictive value for long‐term survival, which may affect the generalizability of the correlation analysis between lipid A characteristics and clinical parameters. In terms of mechanistic research, the current in vitro and in vivo experiments have only verified the effects of single bacterial genera, such as *Klebsiella* and *Baumannii*, and it is impossible to determine whether microbial changes are the driving factors or accompanying phenomena of tumor progression. Future research needs to integrate metagenomics, metabolomics, and high‐resolution mass spectrometry imaging technologies to construct a multimodal‐driven comprehensive map of tumor‐associated microorganisms, and validate their biological and clinical significance through multicenter cohort studies.

## 3 Conclusion

In this study, we constructed a novel ILM called Nor@DHB, which enables direct MALDI MS‐based analysis of lipid A in tumor tissues for the characterization of bacterial expression patterns within LC tissues. Compared with traditional methods, this newly established intratumoral bacterial analysis system eliminates the need for cultivation, exhibits higher sensitivity, and simultaneously addresses the challenge of poor quantitative capability with MALDI MS. Through direct MALDI MS analysis of LC and its paired adjacent noncancerous tissues, eight lipid A biomarkers closely associated with the pathological progression of LC were identified, representing six types of bacteria. Both in vitro and in vivo validation results have also confirmed the reliability of this newly developed method. In summary, by directly analyzing bacteria within LC tissues, this method can reveal the complex relationship between LC and microorganisms. This is of great significance for understanding the LC pathogenesis and progression, as well as regulation of the host immune system. The identified microorganisms closely related to LC pathology may serve as potential biomarkers and new therapeutic targets for this disease, providing a scientific basis for the development of more effective LC treatment strategies. The application of this method may also be gradually extended its application to other cancer types.

## 4 Experimental Section

### Samples Collection and Preparation

The tissue samples were provided by the Biobank of Shanghai Chest Hospital, with detailed information presented in Table S1 (Supporting Information). LPS extraction from the tissues was performed using a commercial kit (EX1740, Solarbio) and stored at −80 °C for future use. The study was conducted in compliance with the Declaration of Helsinki, and the research protocol was approved by the Medical Ethics Committee of Shanghai Chest Hospital (Approval Number: KS22025). The *Klebsiella* and *Baumannii* strains used for functional validation were provided by the Microbiology Group of the Clinical Laboratory, Shanghai Chest Hospital. The bacteria were heat‐inactivated by boiling at 95 °C for 10 min and then cooled to room temperature prior to use.

### IHC and mNGS

For IHC, LC, and peri‐tumor tissues were fixed in paraformaldehyde for 12 h and embedded in paraffin. After that, KeyGEN One‐Step IHC Assay (KeyGen BioTECH: KGOS60) were used to stain 5 µm thickness through the anti‐LPS antibody (HM6011, Hycult). Sequencing service were provided by Genekinder Medicaltech Co., Ltd (Shanghai, China).

### Functional Validation

A549 cell lines were cultured in Dulbecco's modified eagle's medium (Hyclone, Beijing, China) with 10% fetal bovine serum and 1% penicillin‐streptomycin at a 37 °C cell culture incubator with 5% CO_2_. In functional experiments, heat inactivated bacteria was used at a final concentration of 1 × 10^8^ CFUs mL^−1^. For viability analysis, 1 × 10^6^ cells were subjected to starvation and treated with bacteria in a culture dish. Cell counts were then conducted at 12, 24, and 48 h, respectively. For the colony formation assay, 500 cells were treated with bacteria and incubated for a period of 7 days. Following this, the colonies were washed twice with phosphate buffered saline (PBS) and fixed using 95% ethanol. After air‐drying, the cells were stained with 0.1% crystal violet for a duration of 10 min. Finally, a photograph of the cell colonies was taken. A549 cells were incubated in vitro under diverse conditions for a period of 4 h specifically for animal experiments. Subsequently, male C57BL/6 mice were subcutaneously injected with 5 × 10^6^ cells suspended in 100 µL of PBS. The dimensions of tumor growth were documented, and the mice were euthanized 2 weeks later (Approval Number: KS24005).

### MALDI MS Analysis

Nor@DHB was synthesized via a neutralization reaction between DHB (≥99% purity, Sigma‐Aldrich) and Nor (≥98% purity, Selleck Chemicals). Briefly, Dissolve 10 mg of DHB and an equimolar amount of Nor in 1 mL of acetonitrile:water (1:1 v/v) containing 0.1% trifluoroacetic acid). Vortex the mixture for 5 min to ensure complete dissolution. The morphology of Nor@DHB was confirmed by scanning electron microscope. Before MALDI MS analysis, 1 µL of mixture containing analytes and matrix solution was spotted onto the sample plate and dried at room temperature for 1 h. Spectra were collected using the MALDI MS in the reflectron mode. Ionization was achieved by a 337 nm N_2_ laser, and spectra were calibrated using external standards. Laser energy was set to 80 µJ pulse^−1^. Spectral accumulation involved 2 laser shots per sampling point, with a total of 100 accumulations. To enhance spectral quality, an ion gating channel was configured to exclude low‐mass ions below 1000 Da from interfering with the signal. During laser irradiation, prolonged exposure to fixed sampling points was avoided to improve the reproducibility of the MALDI method.

### Statistical Analysis

Data were expressed as mean ± SD. Differences between the groups were analyzed using the student's *t*‐test. A *P* < 0.05 was considered statistically significant. Logistic Regression machine learning method was chosen as the primary classification method due to its interpretability and suitability for binary outcomes (tumor vs adjacent nontumor tissues). Lasso was employed to address multicollinearity among lipid A peaks and to identify a sparse biomarker panel. This step reduced dimensionality while retaining predictive power, enhancing model generalizability. In the parameter optimization process, the regularization factor (*λ* = 1.0) was selected via grid search with twofold cross‐validation on the training set. Other parameters (penalty type = L2, tolerance = 0.0001) were fixed based on computational stability. For the Lasso model, the optimal *λ* was determined by minimizing cross‐validated mean squared error, balancing variable selection and prediction accuracy. Twofold Cross‐Validation: Adopted to maximize sample utilization given the moderate dataset size. Each fold retained class distribution balance to avoid bias. Training halted if validation AUC did not improve by >10% over the test set, ensuring robustness against overfitting. Data Splitting: 15% of data were randomly reserved as a test set (stratified by outcome to maintain class proportions). The remaining 85% formed the training/validation pool for cross‐validation. AUC was prioritized due to its insensitivity to class imbalance and clinical relevance for diagnostic models. The Hosmer and Lemeshow goodness‐of‐fit test was used to assess the fit of the logistic regression model. The R version used was 4.2.3, and the versions of the main packages involved were: pROC 1.18.5, rms 6.7.1, forestploter 1.1.2, ResourceSelection 0.3.6, dplyr 1.1.4, and MASS 7.3.60.

## Conflict of Interest

The authors declare no conflict of interest.

## Supporting information



Supporting Information

## Data Availability

The data that support the findings of this study are available from the corresponding author upon reasonable request.
