# Supplementary material for: A Novel nor@DHB Matrix for Direct Microbial Analysis in Lung Cancer Tissues
Source: Adv Sci (Weinh). 2025 Jun 25;12(35):e04038. doi: 10.1002/advs.202504038 (PMC12463040; doi:10.1002/advs.202504038)
Supplement: Supplementary file 1 — Supporting Information [file ADVS-12-e04038-s001.docx]

Supporting Information

A Novel nor@DHB Matrix for Direct Microbial Analysis in Lung Cancer Tissues

Liang Shan^1#^, Xin Xu^2#^, Lin Huang^2#^, Dan Li^1^, Yiran Deng^1^, Xiangfei Xue^2^, Susu Guo^1^, Yiman Huang^2^, Xiao Zhang^2^, Yongchun Yu**^2^**, Lifang Ma^1*^, Kun Qian^3*^, Jiayi Wang^1,2**^

1. Department of Clinical Laboratory, Shanghai Chest Hospital, Shanghai Jiao Tong University School of Medicine

2. Shanghai Institute of Thoracic Oncology, Shanghai Chest Hospital, Shanghai Jiao Tong University School of Medicine

3. State Key Laboratory for Oncogenes and Related Genes School of Biomedical Engineering, Institute of Medical Robotics and Med-X Research Institute, Shanghai Jiao Tong University


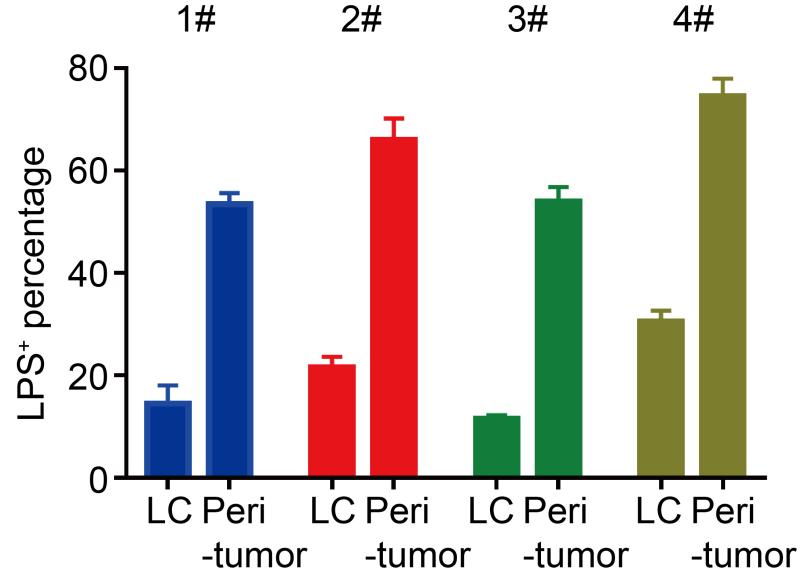


**Supplementary Fig. 1:** LPS^+^ cell percentage.


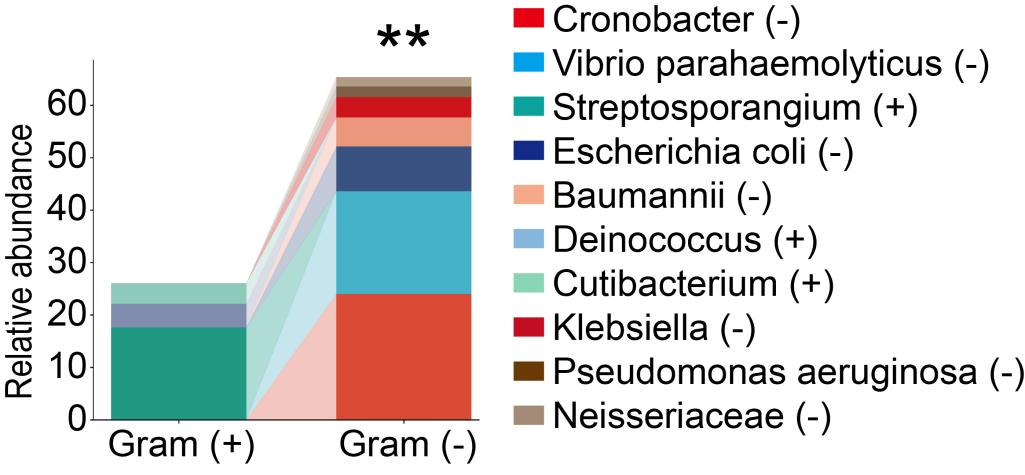


**Supplementary Fig. 2:** Comparison of the abundance of gram-negative and positive bacteria at the genus level in LC, ***P* < 0.01.


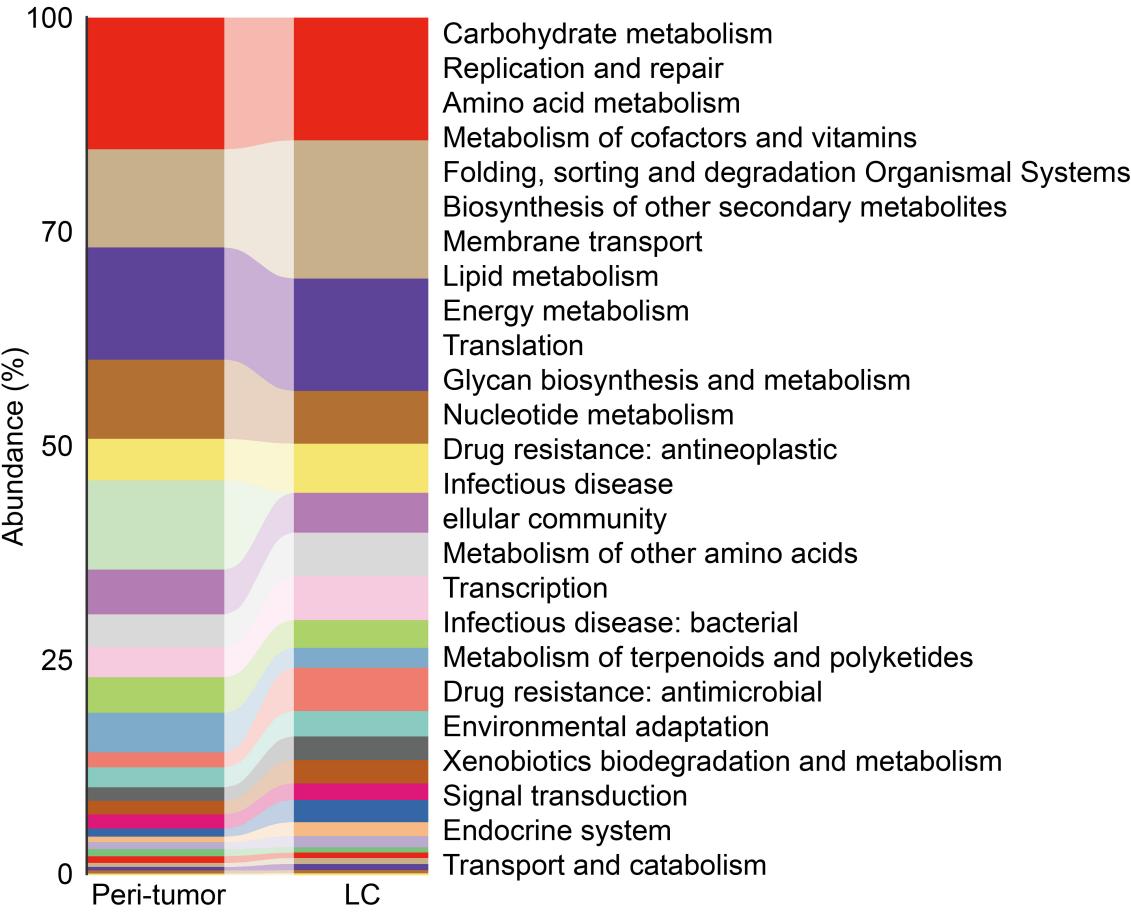


**Supplementary Fig. 3:** Differential bacteria in the KEGG analysis.


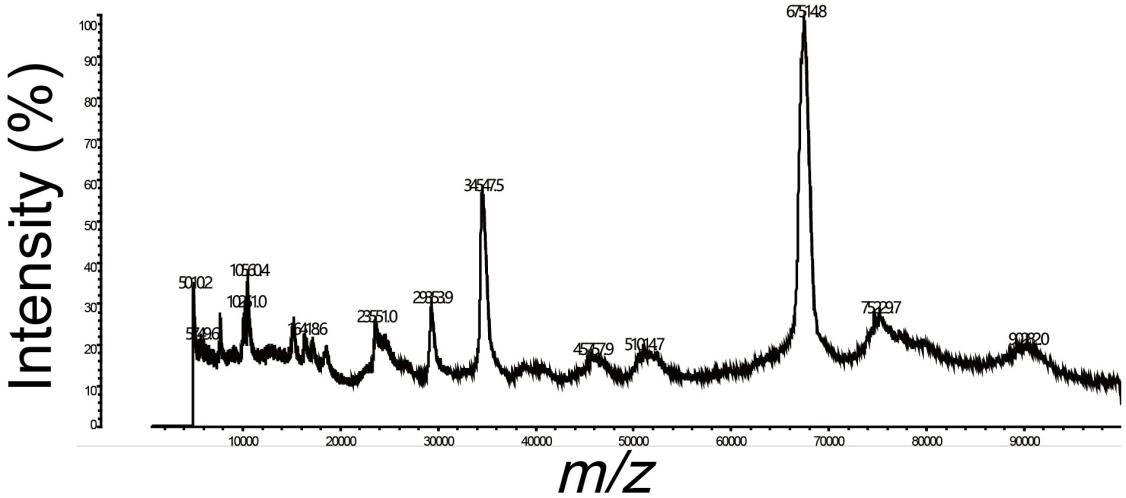


**Supplementary Fig. 4:** The spectra of LC tissues without LPS extraction.


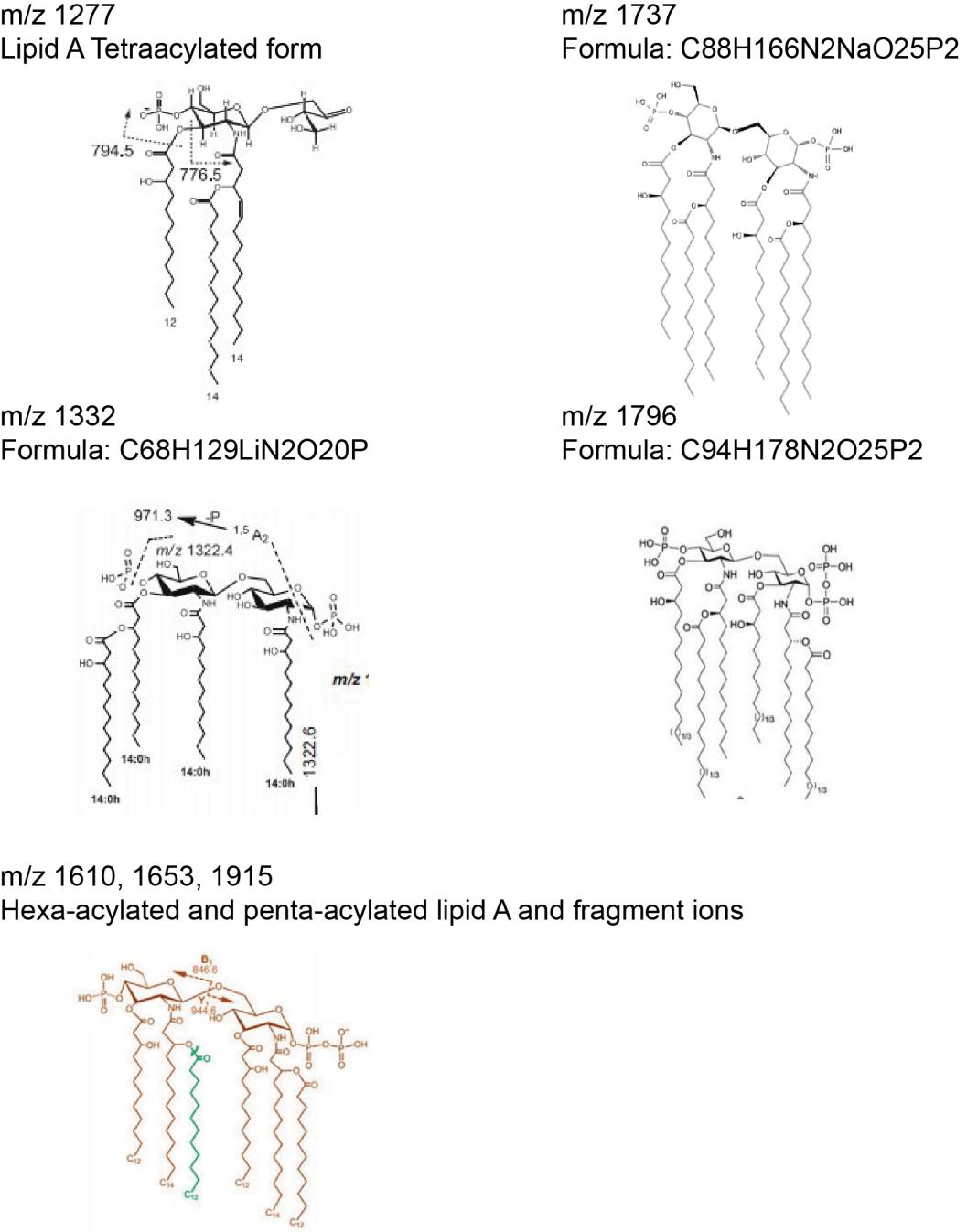


**Supplementary Fig. 5:** The structures of several characteristic lipid A molecules.


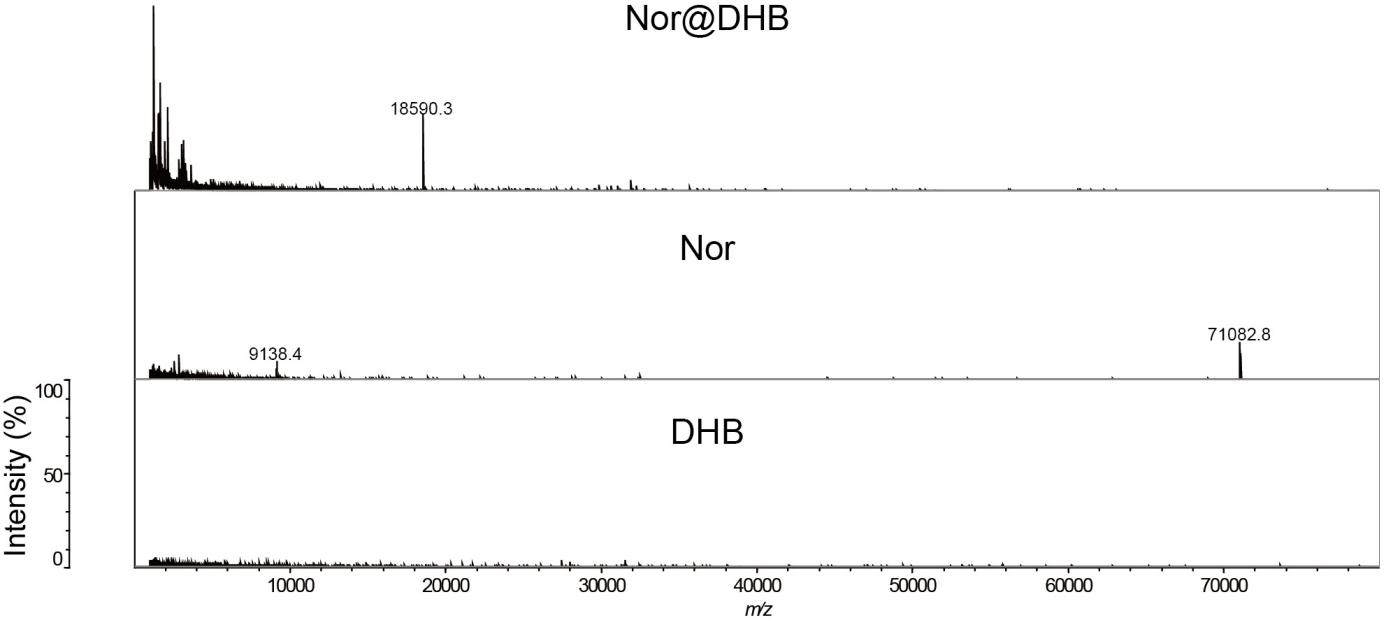


**Supplementary Fig. 6:** The spectra of LPS extracts from LC tissues across a broad mass range.


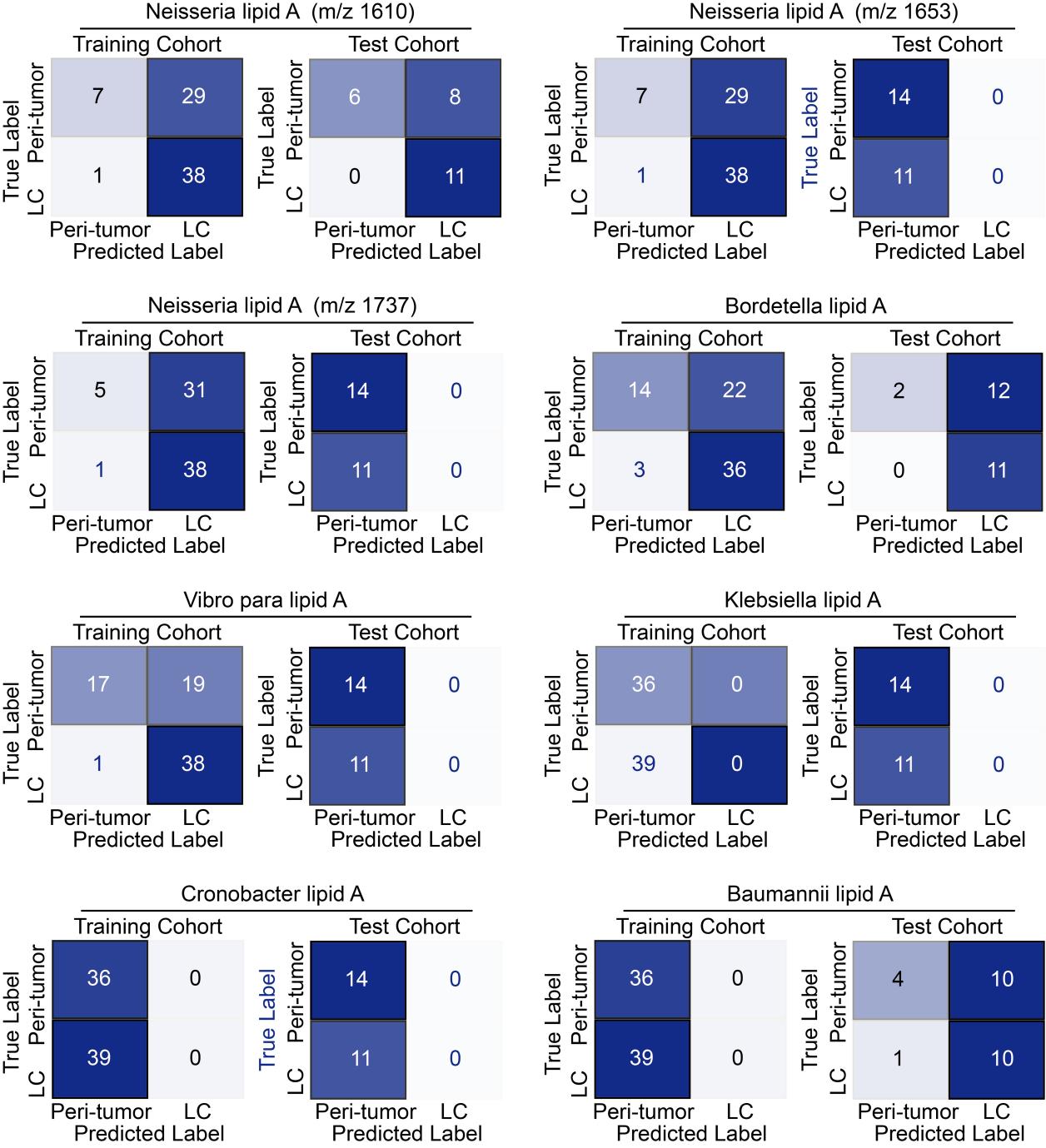


**Supplementary Fig. 7:** Use of the lipid A signals for LC and peri-tumoral tissue classification through the machine learning confusion matrix.


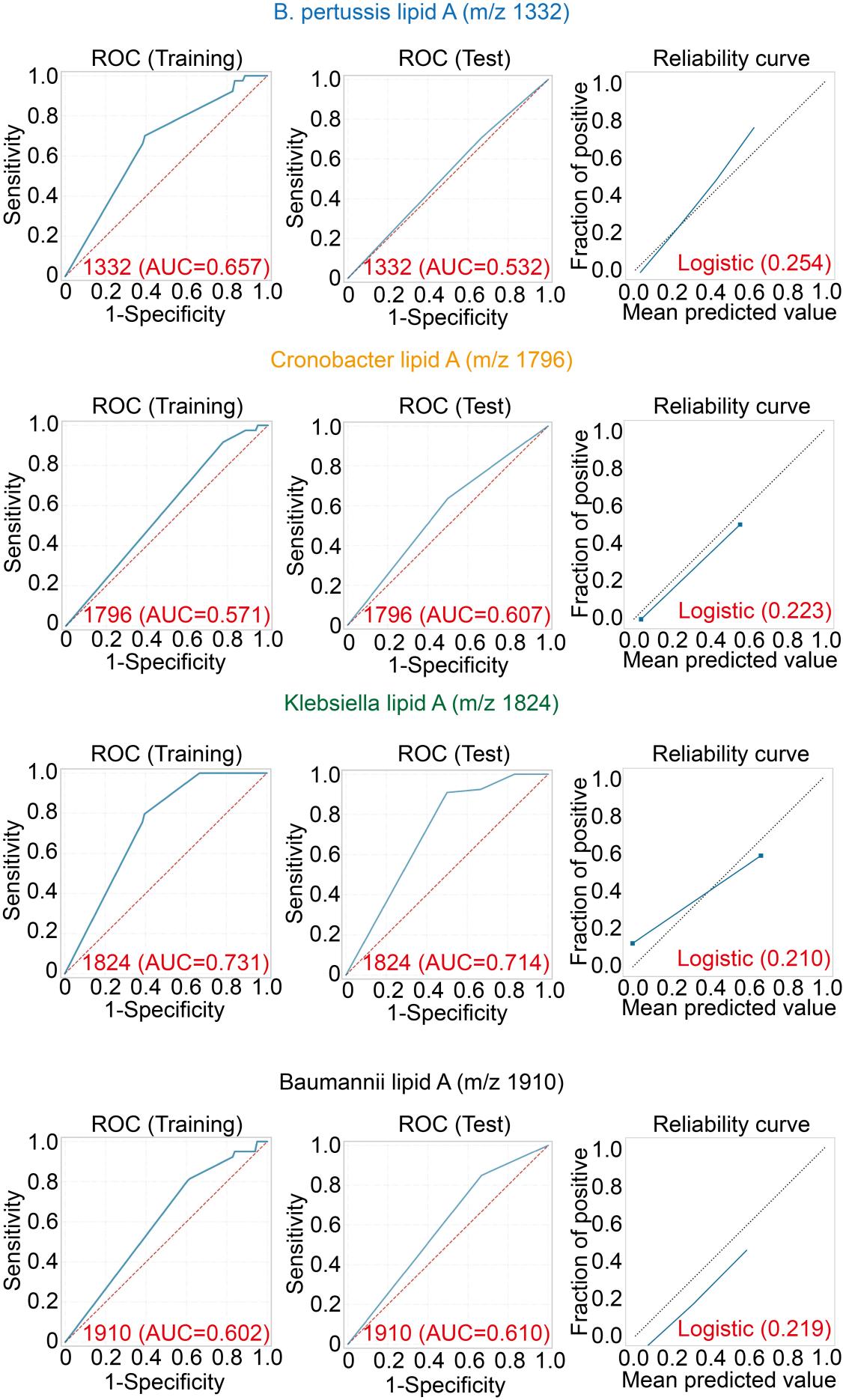


**Supplementary Fig. 8:** Validity (ROC) and reliability analysis of the machine-learning models.


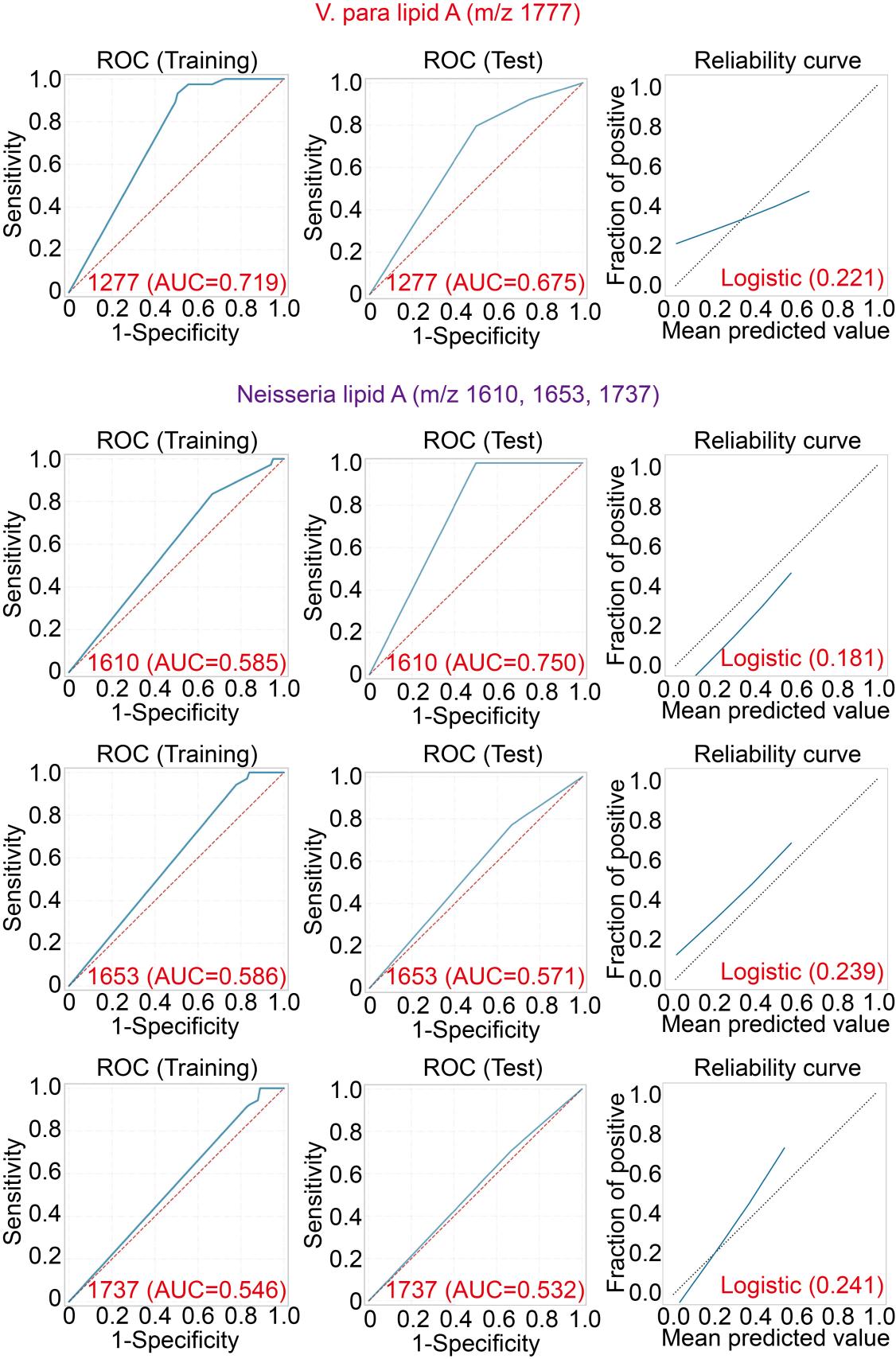


**Supplementary Fig. 9:** Validity (ROC) and reliability analysis of the machine-learning models.

**
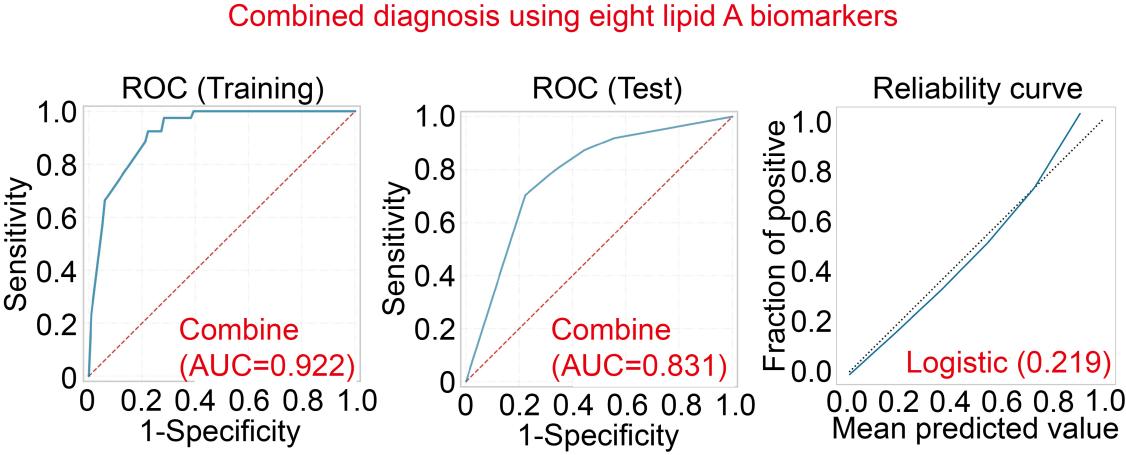
**

**Supplementary Fig. 10:** Validity (ROC) and reliability analysis of the machine-learning models.

**Supplementary Note 1:** Clinicopathologic features.

| No. | Gender | Ages | Cancer type | Stage | T | N | M | Smoke | Tumor size (cm) |
| --- | --- | --- | --- | --- | --- | --- | --- | --- | --- |
| 1 | male | 76 | LUAD | 2 | 3 | 3 | 0 | 1 | 3.4*2.5*2.2 |
| 2 | male | 57 | LUAD | 2 | 2 | 2 | 0 | 1 | 3.2*2.5*2 |
| 3 | female | 68 | LUAD | 1 | 3 | 3 | 0 | 0 | 3*2*1.5 |
| 4 | male | 45 | LUAD | 2 | 1 | 2 | 0 | 1 | 2*1.5*1 |
| 5 | female | 57 | LUAD | 2 | 2 | 3 | 0 | 1 | 3*2*2 |
| 6 | male | 66 | LUAD | 2 | 3 | 2 | 0 | 1 | 2.5*2*1 |
| 7 | female | 65 | LUAD | 2 | 3 | 2 | 0 | 0 | 4*2.5*2 |
| 8 | female | 67 | LUAD | 2 | 2 | 3 | 0 | 0 | 2.8*1.7*1.5 |
| 9 | male | 77 | LUAD | 2 | 2 | 2 | 0 | 0 | 3.3*2.5*2.2 |
| 10 | male | 63 | LUAD | 2 | 2 | 2 | 0 | 1 | 3.5*1.7*1.1 |
| 11 | male | 78 | LUAD | 1 | 3 | 3 | 0 | 0 | 3.8*3.3*2.7 |
| 12 | male | 60 | LUAD | 2 | 3 | 3 | 1 | 1 | 2.5*2*2 |
| 13 | female | 54 | LUAD | 2 | 2 | 3 | 0 | 1 | 2.2*1.8*1.5 |
| 14 | female | 53 | LUAD | 2 | 1 | 1 | 0 | 0 | 1.6*1*1 |
| 15 | female | 73 | LUAD | 2 | 3 | 2 | 0 | 1 | 3*2*2 |
| 16 | female | 67 | LUAD | 2 | 4 | 3 | 1 | 1 | 6*3*3.5 |
| 17 | female | 52 | LUAD | 2 | 3 | 2 | 0 | 1 | 2.6*2*1.5 |
| 18 | male | 61 | LUAD | 1 | 2 | 3 | 0 | 0 | 2.5*2.5*1.6 |
| 19 | female | 53 | LUAD | 1 | 2 | 3 | 0 | 0 | 2.5*2.5*2 |
| 20 | female | 58 | LUAD | 1 | 2 | 3 | 0 | 0 | 2.8*2.5*1.5 |
| 21 | female | 57 | LUAD | 1 | 2 | 2 | 0 | 0 | 2.4*2.2*1.5 |
| 22 | female | 70 | LUAD | 2 | 3 | 3 | 0 | 1 | 3.8*3.2*2 |
| 23 | male | 67 | LUAD | 2 | 4 | 3 | 0 | 0 | 6.8*6*5 |
| 24 | female | 58 | LUAD | 2 | 2 | 2 | 0 | 1 | 2*1.5*1 |
| 25 | female | 57 | LUAD | 2 | 2 | 2 | 0 | 1 | 2.8*2.5*2 |
| 26 | male | 77 | LUAD | 2 | 4 | 3 | 1 | 1 | 2.8*2.5*1.5 |
| 27 | female | 70 | LUAD | 1 | 3 | 3 | 0 | 0 | 2.5*2.5*2.2 |
| 28 | female | 65 | LUAD | 2 | 3 | 3 | 0 | 0 | 3*3*2 |
| 29 | male | 77 | LUAD | 2 | 2 | 3 | 1 | 1 | 2.5*2.5*2 |
| 30 | female | 82 | LUAD | 2 | 3 | 2 | 0 | 0 | 3.4*2.2*1.5 |
| 31 | male | 74 | LSCC | 2 | 1 | 0 | 0 | 1 | 3.5*3.4*2.5 |
| 32 | male | 52 | LSCC | 1 | 1 | 1 | 0 | 0 | 4*4*3 |
| 33 | male | 66 | LSCC | 2 | 1 | 2 | 1 | 1 | 3*4*3 |
| 34 | male | 51 | LSCC | 1 | 4 | 2 | 0 | 0 | 6*5.8*5 |
| 35 | male | 53 | LSCC | 2 | 2 | 3 | 1 | 0 | 2*1.8*1.8 |
| 36 | male | 54 | LSCC | 2 | 4 | 2 | 1 | 1 | 8*6*6 |
| 37 | male | 53 | LSCC | 2 | 2 | 3 | 1 | 1 | 4.2*4*3.5 |
| 38 | male | 60 | LSCC | 2 | 1 | 0 | 0 | 0 | 4*3.5*3.5 |
| 39 | male | 58 | LSCC | 2 | 3 | 2 | 0 | 1 | 3.5*3*2.5 |
| 40 | male | 76 | LSCC | 2 | 3 | 3 | 1 | 1 | 2.9*2.5*2.4 |
| 41 | male | 64 | LSCC | 2 | 1 | 2 | 0 | 1 | 4*3*3 |
| 42 | male | 67 | LSCC | 2 | 4 | 2 | 1 | 1 | 5*5*4 |
| 43 | male | 50 | LSCC | 2 | 4 | 3 | 0 | 0 | 6.1*5.5*4.6 |
| 44 | male | 86 | LSCC | 1 | 1 | 1 | 0 | 0 | 4.8*4.5*4.3 |
| 45 | male | 67 | LSCC | 1 | 2 | 2 | 1 | 0 | 6.5*5*4 |
| 46 | male | 63 | LSCC | 2 | 2 | 1 | 0 | 1 | 4.5*4*4 |
| 47 | male | 54 | LSCC | 2 | 1 | 0 | 0 | 1 | 3.1*3*3 |
| 48 | male | 59 | LSCC | 1 | 1 | 1 | 0 | 0 | 4.5*4*4 |
| 49 | male | 75 | LSCC | 2 | 2 | 0 | 1 | 0 | 2.8*2*1.5 |
| 50 | female | 67 | LSCC | 1 | 1 | 2 | 0 | 0 | 2.8*2*1.6 |

**Supplementary Note 2:** The *m/z* of lipid A detected by MALDI-MS and the bacterial species they represents.

| Name | *m/z* of lipid A | | | | | | | | | |
| --- | --- | --- | --- | --- | --- | --- | --- | --- | --- | --- |
| Pseudomonas aeruginosa | 1430 | 1446 | 1616 |  |  |  |  |  |  |  |
| Klebsiella | 1188 | 1398 | 1426 | 1796 | 1840 | 1891 | 1971 | 2063 | 2079 |  |
| Baumannii | 1728 | 1824 | 1910 | 2033 |  |  |  |  |  |  |
| Escherichia coli | 1188 | 1340 | 1370 | 1376 | 1737 | 1768 | 1886 | 1928 |  |  |
| Pseudomonas aeruginosa | 1192 | 1376 | 1404 | 1447 | 1463 | 1617 | 1633 |  |  |  |
| Bordetella | 1332 | 1478 | 1559 |  |  |  |  |  |  |  |
| Vibrio parahaemolyticus | 1277 | 1256 | 1293 | 1357 | 1373 | 1458 | 1630 | 1646 |  |  |
| Porphyromonas gingivalis | 1372 | 1448 | 1608 | 1688 | 1692 | 1772 | 2063 |  |  |  |
| Pandoraea pulmonicola | 1098 | 1178 | 1296 | 1376 | 1457 | 1522 | 1537 | 1602 | 1683 |  |
| Neisseria | 1338 | 1555 | 1610 | 1653 | 1733 | 1737 | 1792 | 1856 | 1915 | 2038 |
